# Towards Clinical Diagnoses: Classifying Alzheimer's Disease Using Single fMRI, Small Datasets, and Transfer Learning

**DOI:** 10.1002/brb3.70427

**Published:** 2025-03-19

**Authors:** Samuel L. Warren, Ahmed A. Moustafa, for the Alzheimer's Disease Neuroimaging Initiative

**Affiliations:** ^1^ School of Psychology, Faculty of Society and Design Bond University Gold Coast Australia; ^2^ Department of Human Anatomy and Physiology, the Faculty of Health Sciences University of Johannesburg Johannesburg South Africa

**Keywords:** Alzheimer's disease (AD), clinical diagnoses, deep learning, functional Magnetic Resonance Imaging (fMRI), model usability, transfer learning

## Abstract

**Purpose:**

Deep learning and functional magnetic resonance imaging (fMRI) are two unique methodologies that can be combined to diagnose Alzheimer's disease (AD). Multiple studies have harnessed these methods to diagnose AD with high accuracy. However, there are difficulties in adapting this research to real‐world diagnoses. For example, the two key issues of data availability and model usability limit clinical applications. These two areas are concerned with problems of accessibility, generalizability, and methodology that may limit model adoption. For example, fMRI deep learning models require a large amount of training data, which is not widely available. Contemporary models are also not typically formatted for clinical data or created for use by non‐specialized populations. In this study, we develop a deep‐learning fMRI pipeline that addresses some of these issues.

**Method:**

We use transfer learning to address problems with data availability. We also use semi‐automated and single‐image techniques (i.e., one fMRI volume per participant) to make a model that is usable for non‐specialized populations. Our model was initially trained on 524 participants from the Autism Brain Imaging Data Exchange (ABIDE; Autism and controls). Our model was then transferred and fine‐tuned to a small sample of 64 participants from the Alzheimer's Disease Neuroimaging Initiative (ADNI; AD and controls).

**Findings and Conclusion:**

This transfer learning model achieved an AD classification accuracy of 77% and outperformed the same model without transfer learning by approximately 30%. Accordingly, our model showed that small AD samples can be accurately classified in a clinically friendly manner.

## Graphical abstract

An outline of our transfer learning pipeline that is initially trained on an autism spectrum disorder (ASD) dataset and then fine‐tuned to an Alzheimer's disease (AD) classification task.

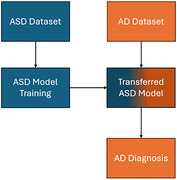

## 1 Introduction

Alzheimer's disease (AD) diagnoses require significant innovation to accurately detect the disease and improve individuals’ quality of life (Liss et al. 2021). For example, it is estimated that improvements in early detection and diagnostic accuracy could result in new treatments and interventions that can combat AD (Porsteinsson et al. 2021). Consequently, there is a sizable literature that examines cutting‐edge methods for AD diagnosis. Functional magnetic resonance imaging (fMRI) and deep learning are two such methods that have been combined to create cutting‐edge AD diagnosis models (Rashid et al. 2020; Warren and Moustafa 2023). While these two methods are not the only point of intrigue (e.g., structural MRI and machine learning methods are equally popular; Ebrahimighahnavieh et al. 2020; Tanveer et al. 2020), fMRI and deep learning are important as they can harness unique functional biomarkers that could be key to diagnosing AD (e.g., brain activation and connectivity; Márquez and Yassa 2019). Multiple studies have shown that fMRI deep learning models can address key areas of AD diagnoses, such as detecting the early stages of the disease, predicting the disorder's development, and accurately diagnosing various stages of the AD continuum (e.g., MCI; Abrol et al. 2019; Ju et al. 2019; Ramzan et al. 2019).

However, there is a clear disconnect between AD diagnoses conducted in research and clinical settings (Chandra et al. 2019). This disconnect is detrimental as it impedes the application of cutting‐edge research methods, like fMRI deep learning models, to clinical AD diagnoses. For example, the *National Institute on Aging* and the *Alzheimer Association* workgroups currently do not consider fMRI methods for clinical diagnoses and only cautiously suggest using some structural neuroimaging methods (Jack et al. 2011, 2018). In contrast, the contemporary research literature uses various structural and functional imaging methods to diagnose AD (R. Li et al. 2022; Márquez and Yassa 2019). This disconnect between research and practice may occur because most cutting‐edge methods are only proof of concepts, are not commonly made with clinical diagnoses in mind, or are held back by methodological problems (e.g., data accessibility and generalizability). It is important to note that some problems with fMRI and deep learning models occur due to logistical and economic problems outside the scope of the brain sciences (e.g., policy, healthcare, and engineering; Constable 2023; Specht 2020). For example, the cost of brain scans and the availability of MRI machines can limit the accessibility of fMRI diagnoses (especially in poorer and remote communities). Nevertheless, these are addressable problems that researchers can tackle. Consequently, there is a critical need to address the current issues with fMRI deep learning models and close the gap between research and clinical AD diagnoses.

In neuroscience research, addressing the two problems of model usability and data availability may be key to making fMRI deep learning models clinically viable. Model usability refers to the ability of a clinician or healthcare worker to operate an fMRI deep learning model. For example, a model will lack clinical usability if it requires specialized computational skills to perform and interpret diagnoses (Valliani et al. 2019). Simply put, most contemporary models may not be clinically viable because they are not made to be operated by non‐specialized populations (e.g., healthcare workers; Teng et al. 2023). Model usability might also be hindered because contemporary models may not be formatted for clinical data or trained on representative samples (i.e., poor generalizability). Some aspects of model usability have been discussed in the literature through the lens of model interpretability (Teng et al. 2023; Valliani et al. 2019). This article builds on this literature and seeks to streamline the use of all aspects of a diagnostic pipeline for clinical settings (e.g., data, preprocessing, analysis, and interpretation). We also focus on streamlining models (e.g., automation) to suit healthcare workers’ skill sets and real‐world diagnostic scenarios (e.g., small clinical settings). Accordingly, in this article, we create an AD classification model that adopts contemporary techniques (e.g., automated methods and single‐image formatting) to address problems with model usability.

Data availability is the second addressable issue that we suggest impedes the clinical viability of fMRI deep learning models. We define data availability as the quantity and accessibility of fMRI data for making deep‐learning classification models. The accessibility of fMRI is important as deep learning models require a large amount of data to classify AD accurately. At the moment, fMRI data is not widely available for AD, which can directly restrict the accuracy and generalizability of deep learning models (Wen et al. 2018). For example, one of the most popular AD datasets—The Alzheimer's Disease Neuroimaging Initiative (ADNI) 2—only contains approximately 709 resting‐state fMRI images (Beckett et al. 2015). These comparatively small AD samples are problematic as they often lead to overfitting. Overfitting is a phenomenon that occurs when a model memorizes a dataset rather than learning generalizable features for diagnoses. An overfitted model will perform nearly perfectly on its training data due to this memorization but will generalize poorly to other conditions (e.g., validation and testing; Smucny et al. 2022). Small samples are vulnerable to overfitting because memorization can easily occur when there is a large disparity between model and dataset size. These problems with data availability directly influence the creation of fMRI deep‐learning models and limit the application of these diagnostic models to small front‐line AD populations (e.g., local clinics, general practices, rural medicine, and small hospitals). In this paper, we apply state‐of‐the‐art techniques (i.e., transfer learning) to increase data availability and create a model that can diagnose a small AD sample.

Some popular and emerging techniques can be applied to fMRI deep learning models to improve model usability and data availability. Specifically, data sharing, data augmentation, regularization (e.g., dropout, weight decay, and early stopping), and transfer learning can combat problems with data availability (X. Li et al. 2019; Santos and Papa 2022; Smucny et al. 2022). Similarly, problems with model usability can be addressed using methods like single‐image formatting (i.e., not segregating an image), automation, and improving data availability (e.g., generalizability; Valliani et al. 2019). For example, data augmentation is a technique that artificially increases a dataset's size by transforming images through functions like rotation, warping, color shifting, and Gaussian blur (Chlap et al. 2021; Nguyen et al. 2020). These altered images are often randomly introduced into a model's training dataset (e.g., half of all images are randomly rotated), thus decreasing overfitting and increasing generalizability. Many of these methods have been used throughout the literature. For example, Alorf and Khan (2022) used data augmentation, Qureshi et al. (2019) used dropout, Abrol et al. (2019) used early stopping, we have previously used an automated preprocessing pipeline (Warren et al. 2024), and data sharing is used throughout the literature (e.g., ADNI; Teng et al. 2023). However, transfer learning and single‐image formatting techniques are not widely used in the combined fMRI, deep learning, and AD literature. These techniques are advantageous for addressing problems of data availability and usability because of their ability to preserve testing data, simplify preprocessing pipelines, and deploy to edge cases (e.g., small clinics and local hospitals). Moreover, transfer learning and single‐image formatting are complementary to other techniques such as data augmentation, regularization, and automation. In this article, we explicitly focus on using transfer learning to improve data availability and single‐image formatting to assist with model usability. Both of these techniques are discussed in more detail in the following sections.

### 1.1 Transfer Learning

**FIGURE 1:**
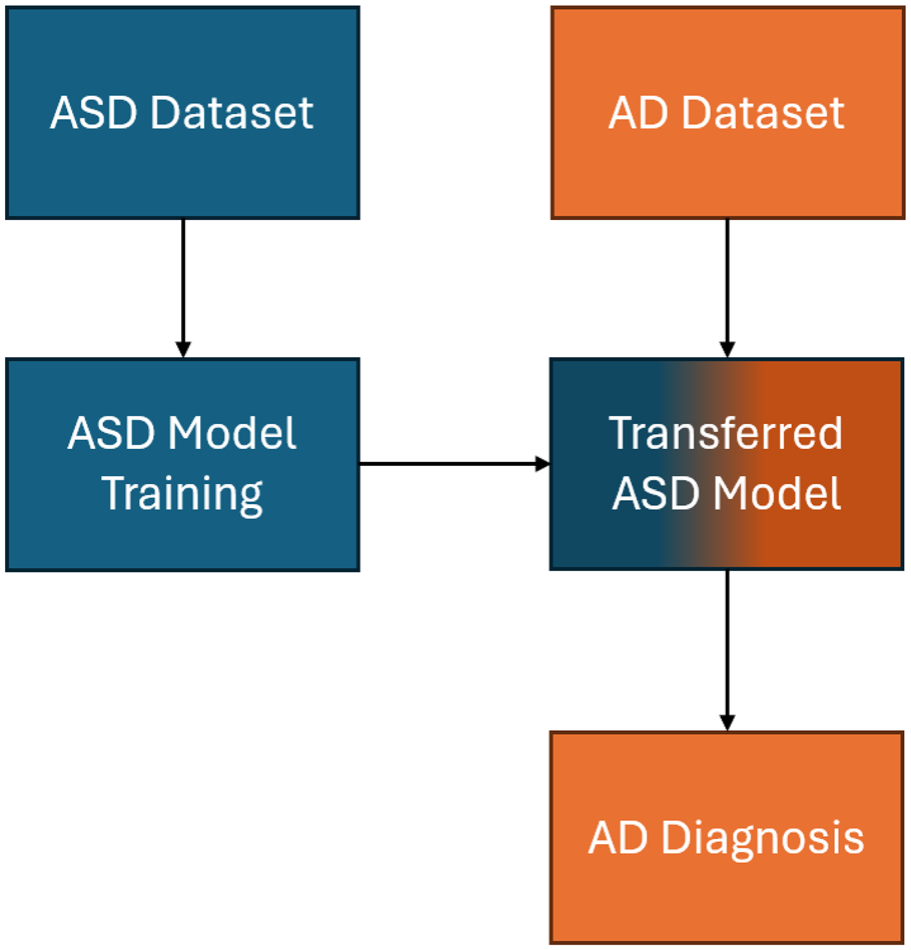
An example transfer learning scenario. An example of transfer learning that trains a model on an autism spectrum disorder (ASD) dataset and then transfers the model to an Alzheimer's disease (AD) diagnosis task. The color blue indicates the ASD model and its knowledge that is then fine‐tuned to an AD classification problem (orange) using an AD dataset.

Transfer learning is a machine learning technique that increases data availability by incorporating task‐adjacent data. Specifically, the technique works on the premise that knowledge from one task can be applied to an adjacent or overlapping problem (Ravishankar et al. 2016). Thus, a transfer learning model will typically learn classification features from one dataset and then transfer that knowledge to a similar classification task on a new dataset (Iman et al. 2023). For example, an AD transfer learning model can increase the size of its dataset by incorporating training data from a similar condition to Parkinson's disease (See Figure 1). There are multiple formats for transfer learning, such as the transfer of features, datasets, or models (Niu et al. 2020). Using pre‐trained models (i.e., model architecture and weights) is one of the most common transfer learning methods used in the AD literature. In these scenarios, researchers take a pre‐existing model (e.g., ResNet) that has been pre‐trained on a non‐AD task (e.g., ImageNet) and apply it to an AD classification problem (Deng et al. 2009; He et al. 2015). For example, a study by Ramzan et al. (2019) used a network known as ResNet18 with pre‐trained weights to classify AD. These pre‐trained models are popular as they are openly available, cutting‐edge, and are generally considered to perform better than most manually trained models. In AD research, the largest benefits of transfer learning are arguably data maximization and generalization. With transfer learning, the additional pre‐trained knowledge can increase the classification ability in small datasets and prevent overfitting (Niu et al. 2020). Moreover, the technique is beneficial for small datasets as the original training data does not need to be disease‐related, meaning that most AD data can be preserved for fine‐tuning or classification (Shanmugam et al. 2022). Accordingly, transfer learning is a powerful technique that can help increase data accessibility and improve the generalizability of small datasets.

In AD research, transfer learning has mostly been used with structural MRI (sMRI) and deep learning. For example, a systematic review by Agarwal et al. (2021) found that only 1 out of 13 relevant studies used fMRI and transfer learning. In contrast, the remaining 12 studies used sMRI or positron emission tomography (PET) and transfer learning for AD classification. This predominantly structural imaging literature has shown that transfer learning can be applied to AD with high effect (Aderghal et al. 2020; Ebrahimi‐Ghahnavieh et al. 2019; Lu et al. 2022). For example, a study by Hon and Khan (2017) found that transfer learning could increase the accuracy of their AD model from 74% to 92%. However, the generalizability of these findings to fMRI is somewhat unknown. At the moment, there are a few pioneering works on fMRI and transfer learning for AD classification. For example, Ramzan et al. (2019) used a pre‐trained model to classify multiple stages of the AD continuum using fMRI data from 138 participants. Their resulting model could perform multiclass AD classification with an accuracy of 98%. L. Li et al. (2022) also classified AD using fMRI and deep transfer learning. However, unlike Ramzan et al., they did not use a pre‐trained model and instead transferred knowledge from an autism spectrum disorder (ASD) model to an AD classification task. This manual training and transfer learning enabled L. Li et al. to use a unique graphical convolutional neural network (GCN) model, perform deep learning classification on fMRI time series, and classify AD with an accuracy of 89%. Accordingly, preliminary research suggests that deep learning can improve data accessibility in AD, fMRI, and deep learning research.

**FIGURE 2:**
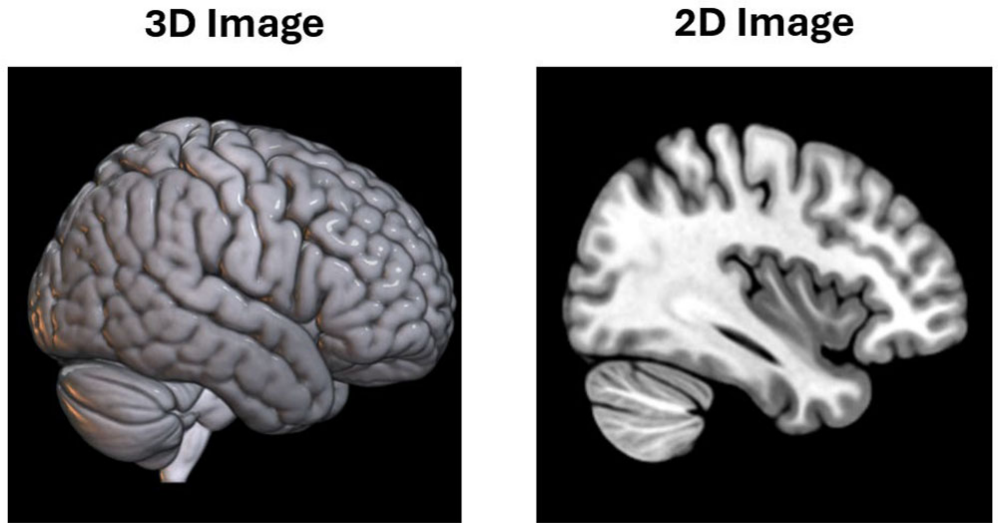
An example of a 3D volume and 2D slice of the same MRI scan. Note: These images were captured or rendered in MRIcroGL using the MNI152 standard. They are sMRI not fMRI (as fMRI are not a good visual example due to their spatial image quality) and only serve as an example of dimensionality.

However, there is still a dearth of research on fMRI, transfer learning, and AD classification. We speculate that the lack of transfer learning application may occur due to the smaller size of the fMRI literature, the shortage of fMRI data, and the dimensionality of fMRI data. For example, fMRI data may lack use with pre‐trained models because it is commonly three‐ or four‐dimensional, whereas almost all pre‐trained models are two‐dimensional (2D). This dimensional restriction can limit the quality of fMRI data (i.e., data loss from dimensional reduction) and could be preferential towards structural mediums (e.g., fMRI volumes tend to work easier with pre‐trained models than time series). See Figure 2 for an example of different dimensionalities. In this study, we address these limitations by creating an AD classification model that works with transfer learning, three‐dimensional (3D) techniques, and a small fMRI dataset. In turn, we create a model that can work with small samples, avoid overfitting, and reduce data loss from dimensional reduction. Consequently, we assess the viability of transfer learning methods in fMRI and AD research and address issues with data accessibility.

### 1.2 Single‐Image Formatting

Like many cutting‐edge techniques, transfer learning lacks clinical use and can negatively affect clinical usability. One key problem is that data is often dimensionally reduced to fit 2D pre‐trained models (as discussed above). The reason for this dimensional reduction is due to the lack of higher dimensional pre‐trained models. There are some 3D pre‐trained models; however, they are for video and have not been widely applied to fMRI (Han et al. 2021). As a result, almost all fMRI deep learning studies will use a 2D pre‐trained model when performing transfer learning for AD classification. A typical deep learning study will achieve dimensional reduction by taking a 3D preprocessed fMRI image and then slicing it into a series of 2D images (e.g., Sarraf et al. 2019). This slicing approach is advantageous as it can reduce the complexity of the model (e.g., only 2D convolutions are required) and reduce the computational resources required for classification. However, dimensional reduction can also result in data loss and additional preprocessing steps (Singh et al. 2020). In turn, data slicing requires specialized skills that may not be available in clinical practices and may result in losing key imaging features. The usage of 3D images also allows for a streamlined pipeline, retaining the same image format from the MRI scanner to the classification model. Accordingly, we argue that using a single 3D fMRI per participant could result in a transfer learning model that is more usable for clinicians.

Some fMRI deep learning models use a single image per participant. For example, Duc et al. (2020) classified AD using a 3D‐convolutional neural network (3D‐CNN) with preprocessed fMRI volumes as input. Their resulting model could classify AD from controls with an accuracy of approximately 85%. It is difficult to compare the accuracy of 2D and 3D models due to the difference in feature extraction and architectures. Nevertheless, it is generally considered that 3D models can be advantageous due to their higher dimensional features (i.e., they can capture more complex relationships). In the wider literature, some models pair 3D images with transfer learning. For example, Maqsood et al. (2019) used 3D sMRI and transfer learning to classify AD with a multiclass accuracy of approximately 93%. Basaia et al. (2019) also trained a model on 3D sMRI images that were privately collected or acquired from the ADNI database. Their resulting model could classify AD with an accuracy of approximately 98%. To our knowledge, there are no 3D models that diagnose AD using transfer learning and fMRI. This lack of research is potentially due to the absence of pre‐trained 3D models and the shortage of fMRI data discussed above. Nevertheless, the sMRI literature indicates that single‐image techniques and transfer learning are compatible. In this article, we investigate whether single‐image formatting can generalize to fMRI and enable transfer learning in a clinically viable manner.

### 1.3 The Current Study

In this article, we create a novel deep‐learning fMRI model for AD classification that addresses common problems with clinical viability. Specifically, we combine contemporary techniques— such as automated methods, single image formatting, transfer learning, and data augmentation— to counteract problems with data access and model usability. These techniques aim to address common clinical characteristics that clash with current model designs, such as having small datasets, one brain scan per patient, or non‐specialized skills (e.g., no data scientists or neuroscientists). This combination of transfer learning and single‐image formatting approaches is highly novel and actively helps to solve problems with clinical viability by streamlining analyses and maximizing data. We create our model from scratch using a 3D‐CNN and data from the Autism Brain Imaging Data Exchange (ABIDE) and ADNI initiatives. To our knowledge, no prior study has sought to classify AD using a single 3D fMRI per participant, deep learning, and transfer learning. Our research questions are as follows:

**R1**: Can transfer learning and single fMRI images be used to classify AD with a small sample?
**R2**: Does our model achieve a higher classification accuracy with transfer learning than without transfer learning?

Based on these research questions, we hypothesize that it will be possible to classify AD using transfer learning, 3D‐fMRI volumes, and a small sample (Hypothesis 1). We also hypothesize that our model will achieve a higher accuracy with transfer learning than without (Hypothesis 2). The following sections detail our model methods, findings, and subsequent conclusions.

## 2 Methods

### 2.1 Participants and Data

The data for this study was acquired from ABIDE and ADNI, two online research databases (Cameron et al. 2013; Weiner et al. 2017). The data from ABIDE is publicly available and comes preprocessed. Our ABIDE sample contained 524 (*N* = 524) resting‐state fMRI for 284 control and 240 ASD participants. Information about this ABIDE dataset's demographics and preprocessing pipelines has been previously discussed (Warren et al. 2024). The data from ADNI is also freely available but requires application and approval. Our ADNI sample contained 64 (*N* = 64) raw resting‐state fMRI for 32 participants with AD and 32 cognitively normal (CN) controls. The data was obtained from the second ADNI cohort (ADNI2; Beckett et al. 2015). Our participants were randomly selected from the larger ADNI2 sample to simulate a small sample and reduce selection biases. Random selection occurred using a random number generator based on an index of participants’ first fMRI scans (most participants had multiple scans, but only the first was used throughout the study). During participant selection, the AD and control groups were also balanced to assist with model stability. The demographics for our ADNI sample are outlined in Table 1.

**TABLE 1:** ADNI sample descriptive statistics split by diagnosis group.

Group	Count	Age	Education	Sex (F/M)	MMSE
CN	32	75.8 (7.4)	16.6(2.0)	20/12	28.7(1.4)
AD	32	72.5 (7.3)	15.7(3.0)	18/14	22.7(2.5)

*Note*: Numerical variables are displayed as means with standard deviations in brackets, and continuous variables are reported as frequencies.

Abbreviations: AD = Alzheimer's disease, CN = cognitively normal, F = Female, M = Male, and MMSE = mini‐mental state examination

### 2.2 fMRI Preparation

**FIGURE 3:**
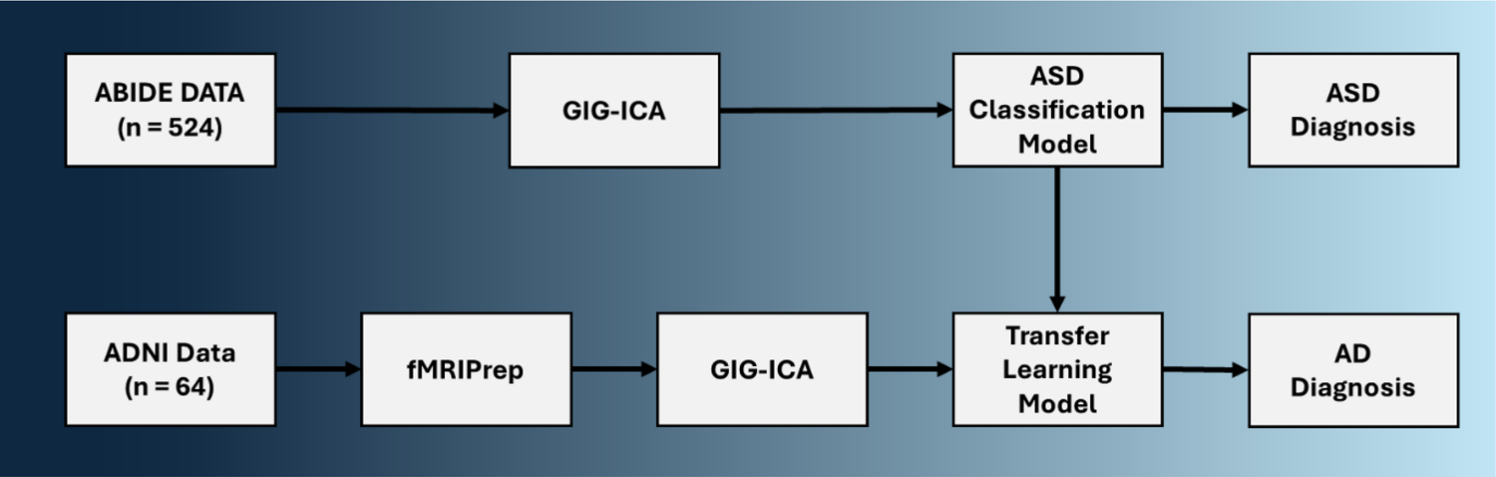
An overview of our transfer learning pipeline. ASD diagnoses were previously performed in a previous article (Warren et al. 2024). fMRIPrep is the program used for preprocessing and a GIG‐ICA is the method used for feature extraction. The ABIDE data came preprocessed and did not require the use of fMRIPrep.

The ABIDE and ADNI samples required preparation before being used in our classification model (see Figure 3). The ABIDE sample is preprocessed and only required feature extraction; however, the ADNI sample was acquired as raw functional and structural MRIs and thus required significant preparation. First, the ADNI data was converted from DICOM to NIFTI formatting to be compatible with our preprocessing software. This conversion was performed using dicm2nii in MATLAB R2022a (X. Li et al. 2016). The same software was also used to format all MRI data into Brain Imaging Data Structure (BIDS), which is also required for our preprocessing pipeline. Our BIDS data was then input into fMRIPrep 21.0.2, a fully automated and standardized preprocessing pipeline for fMRI (Esteban et al. 2019). fMRIPrep takes both anatomical (i.e., sMRI) and functional (i.e., fMRI) as input and outputs cleaned fMRI images. We used fMRIprep on a Windows 10 computer using Windows Subsystem for Linux, Docker, and Ubuntu 18.04. The following two sections are provided by fMRIprep and comprehensively outline all key preprocessing steps. Note that these excerpts are copyright‐free and are intended to be added without alteration for the sake of transparency, replication, and standardization. However, some minor alterations have been made, such as defining all acronyms.

### 2.3 Anatomical Data Preprocessing

A total of 1 T1‐weighted (T1w) image [per participant] was found within the input BIDS dataset. The T1‐weighted (T1w) image was corrected for intensity non‐uniformity (INU) with N4BiasFieldCorrection (Tustison et al. 2010), distributed with ANTs 2.3.3 (Avants et al. 2008, RRID:SCR_004757), and used as T1w‐reference throughout the workflow. The T1w‐reference was then skull‐stripped with a Nipype implementation of the antsBrainExtraction.sh workflow (from ANTs), using OASIS30ANTs as the target template. Brain tissue segmentation of cerebrospinal fluid (CSF), white matter (WM), and gray matter (GM) was performed on the brain‐extracted T1w using fast (FSL 6.0.5.1:57b01774, RRID:SCR_002823, Zhang et al. 2001). Volume‐based spatial normalization to one standard space (MNI152NLin2009cAsym) was performed through nonlinear registration with antsRegistration (ANTs 2.3.3), using brain‐extracted versions of both T1w reference and the T1w template. The following template was selected for spatial normalization: ICBM 152 Nonlinear Asymmetrical template version 2009c [Fonov et al. (2009), RRID:SCR_008796; TemplateFlow ID: MNI152NLin2009cAsym].

### 2.4 Functional Data Preprocessing

For each BOLD [i.e., fMRI] run per subject (across all tasks and sessions), the following preprocessing was performed. First, a reference volume and its skull‐stripped version were generated using a custom methodology of fMRIPrep. Head‐motion parameters with respect to the BOLD reference (transformation matrices, and six corresponding rotation and translation parameters) are estimated before any spatiotemporal filtering using MCFLIRT (FSL 6.0.5.1:57b01774; Jenkinson et al. 2002). BOLD runs were slice‐time corrected to 1.47 s (0.5 of slice acquisition range 0–2.94 s) using 3dTshift from AFNI [Analysis of Functional NeuroImages] (Cox and Hyde 1997; RRID:SCR_005927). The BOLD time series (including slice‐timing correction when applied) were resampled onto their original, native space by applying the transforms to correct for head motion. These resampled BOLD time‐series will be referred to as preprocessed BOLD in original space, or just preprocessed BOLD. The BOLD reference was then co‐registered to the T1w reference using mri_coreg (FreeSurfer) followed by flirt (from the FMRIB Software Library [FSL] 6.0.5.1:57b01774; Jenkinson and Smith 2001) with the boundary‐based registration (Greve and Fischl 2009) cost‐function.

Co‐registration was configured with six degrees of freedom. Several confounding time series were calculated based on the preprocessed BOLD: framewise displacement (FD), DVARS (see, Afyouni and Nichols 2018), and three region‐wise global signals. FD was computed using two formulations following Power (absolute sum of relative motions; Power et al. 2014) and Jenkinson (relative root mean square displacement between affines; Jenkinson et al. 2002). FD and DVARS are calculated for each functional run, both using their implementations in Nipype (following the definitions by Power et al. 2014). The three global signals are extracted within the CSF, the WM, and the whole‐brain masks. In addition, a set of physiological regressors was extracted to allow for component‐based noise correction (CompCor, Behzadi et al. 2007). Principal components are estimated after high‐pass filtering the preprocessed BOLD time series (using a discrete cosine filter with 128 s cut‐off) for the two CompCor variants: temporal (tCompCor) and anatomical (aCompCor). tCompCor components are then calculated from the top 2% variable voxels within the brain mask.

For aCompCor, three probabilistic masks (CSF, WM, and combined CSF+WM) are generated in anatomical space. The implementation differs from that of Behzadi et al. in that instead of eroding the masks by 2 pixels on BOLD space, the aCompCor masks subtract a mask of pixels that likely contain a volume fraction of GM. This mask is obtained by thresholding the corresponding partial volume map at 0.05, and it ensures components are not extracted from voxels containing a minimal fraction of GM. Finally, these masks are resampled into BOLD space and binarized by thresholding at 0.99 (as in the original implementation). Components are also calculated separately within the WM and CSF masks. For each CompCor decomposition, the k components with the largest singular values are retained, such that the retained components’ time series are sufficient to explain 50 percent of variance across the nuisance mask (CSF, WM, combined, or temporal).

The remaining components are dropped from consideration. The head‐motion estimates calculated in the correction step were also placed within the corresponding confounds file. The confound time series derived from head motion estimates and global signals were expanded with the inclusion of temporal derivatives and quadratic terms for each (Satterthwaite et al. 2013). Frames that exceeded a threshold of 0.5 mm FD or 1.5 standardized DVARS were annotated as motion outliers. The BOLD time series were resampled into standard space, generating a preprocessed BOLD run in MNI152NLin2009cAsym space. First, a reference volume and its skull‐stripped version were generated using a custom methodology of fMRIPrep. All resamplings can be performed with a single interpolation step by composing all the pertinent transformations (i.e., head‐motion transform matrices, susceptibility distortion correction when available, and co‐registrations to anatomical and output spaces). Gridded (volumetric) resamplings were performed using antsApplyTransforms (ANTs), configured with Lanczos interpolation to minimize the smoothing effects of other kernels (Lanczos 1964). Non‐gridded (surface) resamplings were performed using mri_vol2surf (FreeSurfer).

### 2.5 Feature Extraction

**FIGURE 4:**
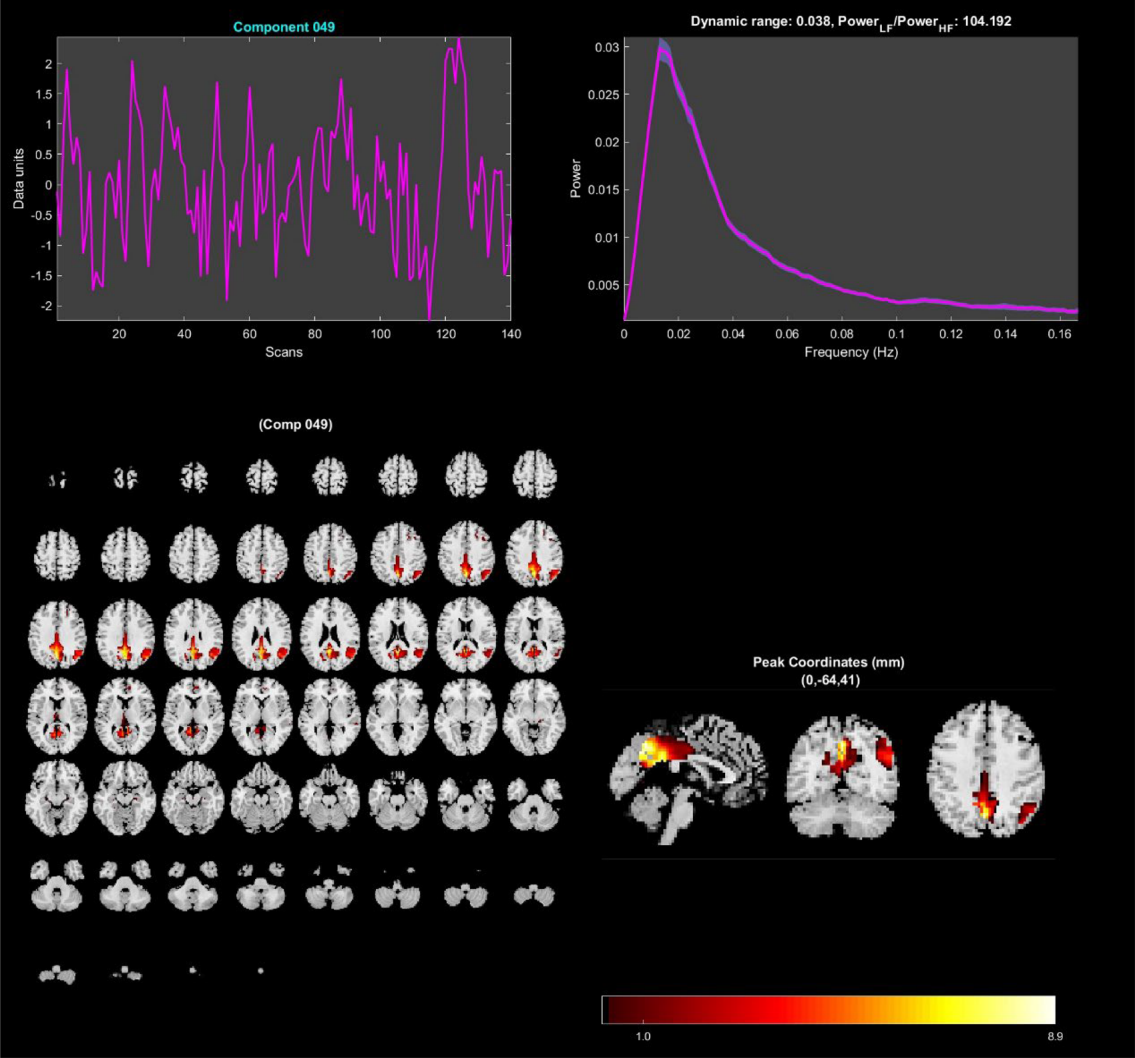
An example independent component from our GIG‐ICA. These images depict the temporal and spatial information for independent component 49. The top two charts show the time course of the fMRI signal while the bottom two images map the brain activation associated with the component. This component shows brain activation in parts of the default mode network.

For feature extraction, we took the preprocessed spatial maps from the ADNI and ABIDE samples and processed them in two separate runs (one for each database). We extracted features for each sample using a spatially constrained group information‐guided independent component analysis (GIG‐ICA; Du and Fan 2013). This GIG‐ICA was conducted in the GITF toolbox via MATLAB R2022a (Rachakonda et al. 2020). We chose to use a GIG‐ICA because it is a data‐driven approach that can obtain brain network features for each participant (e.g., the default mode network). It is also a fully automated approach that can assist with model usability. We used the default mask setting, multi‐objective optimization with reference algorithm, and the NeuroMark template to reliably extract 53 features for each participant (Du et al. 2020). These 53 features comprise the default mode, sensorimotor, cerebellar, sub‐cortical, auditory, cognitive‐control, and visual networks (See Figure 4 for an example of one independent component and Table 2 for details of all 53 components). These methods have been previously discussed in Warren et al. (2024). We chose to use ICA‐derived brain networks as features because they have been shown to classify ASD and AD accurately in the literature (Price et al. 2014; Yang et al. 2010). We stipulate that the similarities in ASD and AD ICA feature‐extraction methods may also benefit transfer learning (for more information, see Warren et al. 2024). Once feature extraction was complete, we had a single preprocessed fMRI that was a spatial map containing seven brain networks for each participant. These spatial maps were then input into our deep‐learning classification model.

**TABLE 2:** The 53 brain‐network features derived from the GIG‐ICA.

Component Number	Dynamic Range	fALFF	Network domain
1	0.024	16.884	Sub‐cortical
2	0.027	20.986	Sub‐cortical
3	0.030	20.273	Sub‐cortical
4	0.026	23.534	Sub‐cortical
5	0.027	21.089	Sub‐cortical
6	0.027	29.801	Auditory
7	0.025	17.090	Auditory
8	0.032	52.242	Sensorimotor
9	0.026	25.330	Sensorimotor
10	0.024	25.510	Sensorimotor
11	0.035	53.852	Sensorimotor
12	0.029	27.369	Sensorimotor
13	0.025	20.364	Sensorimotor
14	0.027	22.971	Sensorimotor
15	0.035	60.858	Sensorimotor
16	0.030	40.909	Sensorimotor
17	0.035	81.523	Visual
18	0.028	31.444	Visual
19	0.031	42.110	Visual
20	0.031	66.826	Visual
21	0.036	63.418	Visual
22	0.027	30.346	Visual
23	0.026	28.950	Visual
24	0.032	68.882	Visual
25	0.027	24.013	Visual
26	0.035	65.683	Cognitive‐control
27	0.027	24.679	Cognitive‐control
28	0.024	23.015	Cognitive‐control
29	0.025	22.449	Cognitive‐control
30	0.032	46.825	Cognitive‐control
31	0.030	34.145	Cognitive‐control
32	0.033	50.906	Cognitive‐control
33	0.036	51.090	Cognitive‐control
34	0.026	24.184	Cognitive‐control
35	0.026	24.578	Cognitive‐control
36	0.030	28.009	Cognitive‐control
37	0.026	16.268	Cognitive‐control
38	0.027	20.381	Cognitive‐control
39	0.024	16.472	Cognitive‐control
40	0.031	37.609	Cognitive‐control
41	0.024	16.108	Cognitive‐control
42	0.023	16.016	Cognitive‐control
43	0.034	61.875	Default‐mode
44	0.040	109.211	Default‐mode
45	0.024	16.015	Default‐mode
46	0.029	38.612	Default‐mode
47	0.022	14.472	Default‐mode
48	0.030	40.012	Default‐mode
49	0.038	104.192	Default‐mode
50	0.023	17.788	Cerebellar
51	0.024	23.652	Cerebellar
52	0.024	22.380	Cerebellar
53	0.026	22.697	Cerebellar

*Note*: fALFF = The ratio of low‐frequency to high‐frequency power.

### 2.6 Deep Learning Model

The architecture and implementation of our baseline ABIDE classification model have been discussed in a previous paper (Warren et al. 2024); however, as a summary, we trained a 3D‐CNN on a binary ASD versus Control classification task. Our 3D‐CNN was a simplified version of C3D's architecture and used hold‐out cross‐validation with an 80% training, 10% validation, and 10% testing split (Tran et al. 2015). The data underwent common normalization and resizing transforms to standardize model inputs. We also used regularisation techniques such as dropout (probability = 0.5), data augmentation (random rotation with probability = 0.5), and early stopping (patience = 15) to help with overfitting. An outline of our architecture is shown in Table 3 and hyperparameters are shown in Table 4.

**TABLE 3:** Model architecture.

Layer	In channels	Out channels	Kernel size	Stride	Padding
Conv3D + LeakyReLU	8	16	6 × 6 × 6	1	0
MaxPool3D	—	—	6 × 6 × 6	2	—
BatchNorm3D	16	—	—	—	—
Conv3D + LeakyReLU	16	32	6 × 6 × 6	1	0
MaxPool3D	—	—	6 × 6 × 6	2	—
BatchNorm3D	32	—	—	—	—
Conv3D + LeakyReLU	32	64	4 × 4 × 4	1	0
MaxPool3D	—	—	4 × 4 × 4	2	—
BatchNorm3D	64	—	—	—	—
Flatten	—	—	—	—	—
Linear + LeakyReLU	64	20	—	—	—
BatchNorm1D	20	—	—	—	—
Dropout	0.5	—	—	—	—
Linear	20	1	—	—	—
Sigmoid	1	1	—	—	—

*Note*: Images underwent normalization and resizing before entering the model, using PyTorch transforms and the dataloader.

**TABLE 4:** Model hyperparameters.

Hyperparameter	ABIDE Model	Transfer learning model
Number of Epochs	100	300
Batch Size	52	63
Random Rotate Probability	0.5	0.7
Random Flip Probability	—	0.7
Dropout	0.5	0.5
Normalize Transform	0.5, 0.25	0.5, 0.25
Resize Transform	61 × 61 × 61	61 × 61 × 61
Learning Rate	0.0001	0.00001
Cross Validation	Hold out 80/10/10	Leave one out 63/1
Early Stopping Patience*	15	—
Optimizer	Adam	Adam
Loss	Binary Cross Entropy (BCE) Loss	BCE Loss

*Note*: Batch size was set to the maximum size of 63 (i.e., the number of participants in the training dataset) to increase model speed. Early stopping was used for the ABIDE model but not the transfer learning model due to LOOCV.

The resulting ABIDE model could classify ASD from controls with an accuracy of 71%, which is competitive with some of the leading models in the literature. To prepare for transfer learning, we took the trained ABIDE model and froze it at its best epoch (i.e., its lowest loss). The best epoch was selected using checkpoints and early stopping. We then saved the frozen model to be used with the ADNI data.

### 2.7 Transfer Learning

The ADNI portion of the model required some changes to the original ABIDE model code before transfer learning could be implemented. First, we had to write a separate data loading script to avoid data leakage and implement a different cross‐validation method. Hold‐out cross‐validation was not appropriate due to our small ADNI sample. Thus, we use leave‐one‐out cross‐validation (LOOCV) to receive an accurate view of our model's classification ability. LOOCV works by training the data on all but one participant, who is the validation sample. This process is repeated until every participant has been the validation sample, and then the accuracy is calculated from the results of all validation runs. We also removed our early stopping function as it is incompatible with LOOCV.

The ADNI data underwent normalization and resizing transforms just like the ABIDE data. Once these changes had been made, we reloaded the frozen ABIDE model and gave it the ADNI data. The model maintained the same dropout (probability = 0.5) and regularization (e.g., batch normalization) as before. However, the learning rate, data augmentation, batch size, and number of epochs were changed to accommodate the new dataset. These hyperparameters are listed in Table 4. When running our transfer learning model, we unfroze all layers to allow for fine‐tuning. This transfer learning paradigm allows the model to update its old knowledge (i.e., original weights from the ABIDE model) with new knowledge relevant to our classification task (ADNI features). The fine‐tuned weights were also cleared and replaced with the original ABIDE weights after every LOOCV run to avoid bypassing the next run through data leakage. Once the transfer learning mode was completed, we ran the same model without transferring learning for comparison. This baseline model was created by running the same code without using the ABIDE weights. The results of these models are discussed in the following sections.

## 3 Results

**FIGURE 5:**
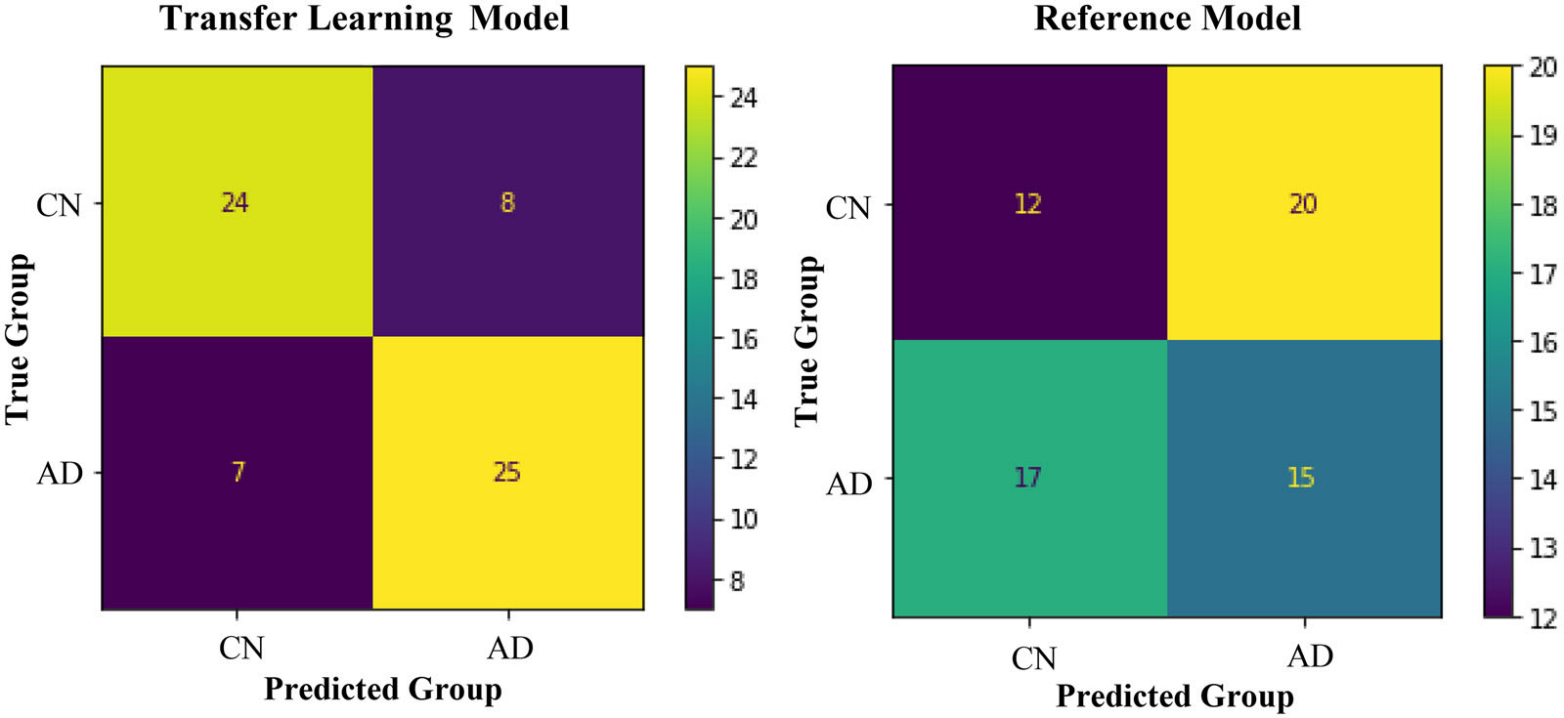
Confusion matrices for the transfer learning and reference model. Note: The colors indicate the frequency participants were classified correctly or incorrectly.

**FIGURE 6:**
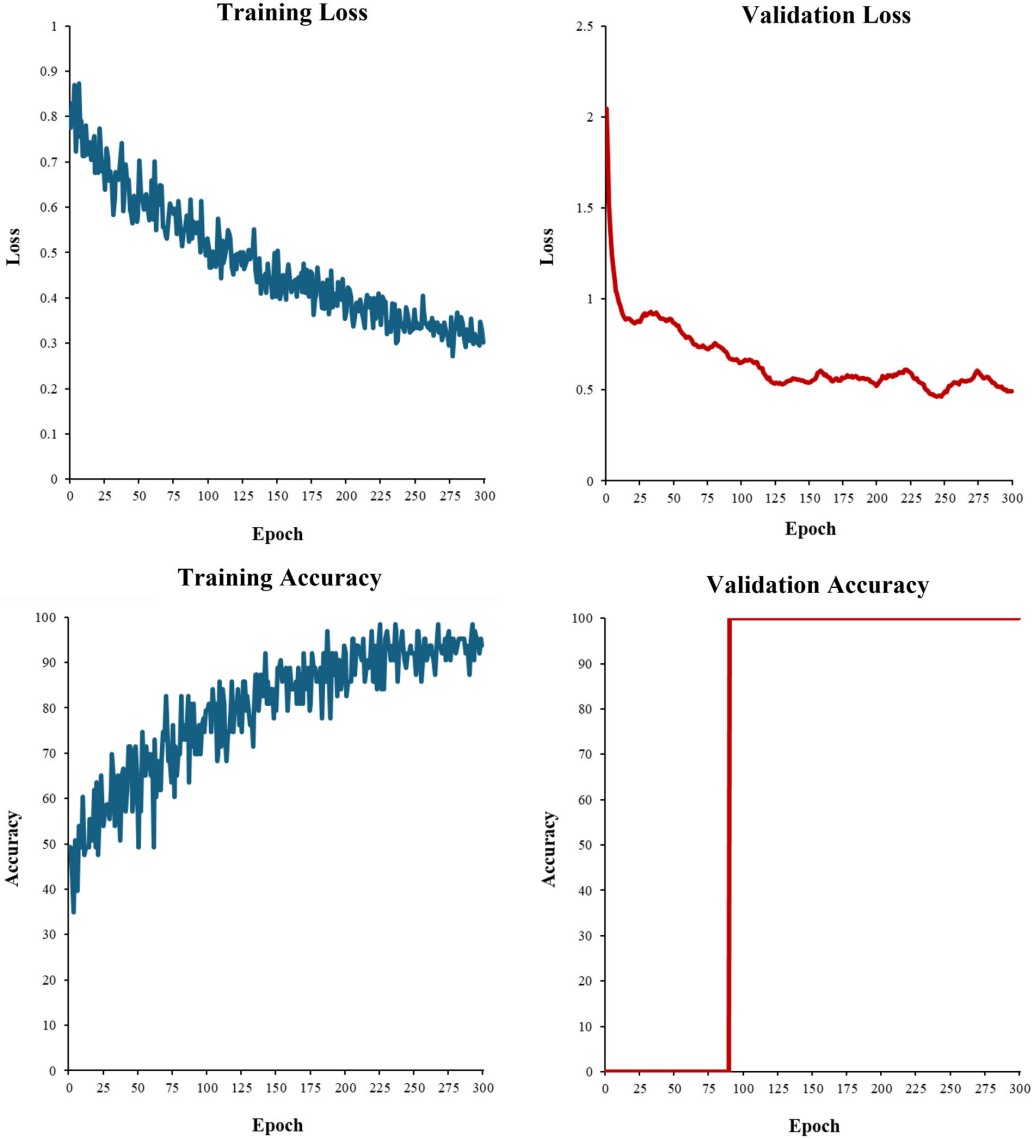
Model Training and Validation Loss and Accuracy. Note: These plots are only an example of one LOOCV iteration. The validation loss and accuracy are mostly superficial statistics due to LOOCV only using one sample; however, they still visualize that the model is learning and generalizing.

Our experiment involved two 3D‐CNN models. These models contained the same hyperparameters and architecture, but only one used transfer learning. We refer to these two models as the transfer learning and reference model (i.e., the same model without transfer learning). The transfer learning model could classify AD from controls with an accuracy of 76.56%, sensitivity of 0.78, specificity of 0.75, and precision of 0.76 (See Figure 5 for specific classification results). For an example of one LOOCV training and validation fold, see Figure 6. Overall, the model had a moderate classification ability, shown by the F1 score of 0.77 and Matthews correlation coefficient (MCC) of 0.53. These results indicate that a small AD sample can be classified using transfer learning, automated techniques, and single‐image formatting. Accordingly, our first research question is answered, and our first hypothesis is accepted.

Next, our reference model could classify AD from controls with an accuracy of 42.19%, sensitivity of 0.47, specificity of 0.38, precision of 0.43, and F1 score of 0.45. These results were very poor, which was reinforced by an MCC of −0.16. These results suggest that AD cannot be classified using our model when using such a small sample, single‐image formatting, and not performing transfer learning. This finding is further reinforced by the 34.37% difference in accuracy between our transfer learning and reference models (see Figure 5). Accordingly, our second hypothesis is also accepted. The implications of the results are further discussed in the following section.

## 4 Discussion

### 4.1 Summary and Literature

In this article, we investigated methods for improving the clinical viability of fMRI and deep learning models. Specifically, we looked at transfer learning and single‐image formatting techniques to address issues with data accessibility and model usability. We aimed to create a model that would work on a small dataset—like a sample from a local clinic— and was semi‐automated. Accordingly, we made a semi‐automated pipeline using fMRIPrep, a GIG‐ICA, and a 3D‐CNN (see Figure 3). Through these methods, we created an AD classification model that could detect the disease with approximately 77% accuracy on only 64 participants with a single fMRI each. The transfer learning in our model was a major contributor to our diagnostic accuracy. Further, the same model without transfer learning performed 34% worse and could not classify AD above chance odds. Consequently, we accepted our hypotheses that transfer learning and single image methods can be used to diagnose a small sample of AD participants (Hypothesis 1) and that our model with transfer learning would perform better than the same model without (Hypothesis 2).

There are few models to compare directly to in the literature. Nevertheless, our best model performed slightly worse than similar models in the literature (approximately 10%–15% lower accuracy). For example, Ramzan et al. (2019) and R. Li et al. (2022) achieved 98% and 89% accuracy, respectively, with fMRI and deep transfer learning models. We speculate that these differences in diagnostic accuracy occurred due to changes in our data, design, aims, and methodology. For example, our small ADNI sample—while purposely chosen to reflect small real‐world samples—will ultimately result in a model with worse generalization (i.e., lower validation accuracy). Nonetheless, some general trends can still be observed across the literature. For example, our 34% increase in accuracy due to transfer learning was very similar to what Hon and Khan (2017) observed in their study. The ability to use a single fMRI volume per participant as input is also consistent with the sMRI literature (Basaia et al. 2019; see section 1.2). Accordingly, our model shows that contemporary techniques, such as transfer learning and single image formatting, can address some of the issues with the clinical viability of fMRI deep learning models.

### 4.2 Limitations

It is important to note that our model had some limitations. One issue is that our model has a lower accuracy than other AD models in the literature. We suspect our lower accuracy comes from our small ABIDE and AD samples. It is widely known that larger data samples can assist with model accuracy, even when using transfer learning. We chose our ABIDE sample due to the similarities between ASD and AD fMRI diagnostic techniques; however, this choice of database did limit our sample size. Next, our methodology may have also limited our model's classification ability. We have previously used a GIG‐ICA and C3D‐based 3D‐CNN and found that they have performed well compared to similar models in the literature (Warren et al. 2024). However, other methods have achieved a higher accuracy than ours and should be considered. We explicitly chose our methods for automation, clinical viability, and not exclusively accuracy, but maybe a more balanced approach is required. There is also an argument to be made about the viability of fMRI when compared to sMRI and other neuroimaging techniques. While this debate is beyond the scope of this article, we highlight the important role of multimodal fMRI and sMRI models, the potential of fMRI for early AD diagnosis (i.e., before significant atrophy), but also acknowledge its significant resource cost and noise associated with fMRI (Warren and Moustafa 2023). These factors should be considered in future research. Lastly, our model was only binary and only focused on late‐stage AD. In the future, we would like to expand our model to incorporate multiple conditions across the AD spectrum (e.g., MCI). However, this approach will require more data and a baseline 3D‐CNN model that can perform multiclass classification. Accordingly, a new AD sample, baseline database (i.e., not ABIDE), and classification architecture will be required. Different deep learning architectures should also be considered, such as vision transformers, autoencoders, and recurrent neural networks; however, the comparison and speculation on better architectures are beyond the scope of this study.

### 4.3 Strengths and Future Directions

Our model also had some significant strengths that should be highlighted. To our knowledge, this is the first model to look at improving the clinical viability of fMRI and deep learning models for AD classification. Accordingly, this model takes the first steps in improving issues with data availability and model usability. Our model also highlighted the usefulness of single‐image formatting and transfer learning techniques in fMRI research. Our increase in classification accuracy from transfer learning is critical to the success of our model and could be pivotal in translating deep learning research methods to small clinical samples. Similarly, the use of automated techniques and single‐image formatting may be key to creating models that are easily operable, accessible, and shareable.

We suggest that future research continue to push for cutting‐edge classification models but also consider the clinical viability of the methods used. We also suggest that future research embrace transfer learning, automated techniques, and single‐image formatting when appropriate. We want to build on this research by investigating other transfer learning methods, such as 3D pre‐trained models (i.e., video models). We would also like to look into methods for strengthening the classification accuracy of models on small samples, such as multivariate models. For example, it would be interesting to see if our current model's classification accuracy could be improved by including clinical data with participants’ fMRI. Lastly, we would like to consider explainable AI (XAI). Clearly, fMRI and deep learning can help researchers classify AD. However, there is a great need to understand how these models perform classification and with what features. Current advancements in XAI may be an avenue for understanding the ability of our model and how it could be improved. For example, it would be beneficial to understand which of our 53 independent components are key for AD classification using perturbation or feature ranking. It would also be valuable to extract our models’ key features using feature visualization (e.g., feature maps). Such XAI methods could be important in understanding what specific biomarkers our model uses to classify AD and help refine fMRI deep learning pipelines.

## 5 Conclusion

This study is a preliminary work investigating the ability of fMRI and deep learning models to be adapted for clinical scenarios. This clinical viability is important as it gives the field a pathway toward real‐world diagnoses and can potentially improve clinical outcomes. For example, such innovations could result in AD being detected earlier and more quickly, thus improving individuals’ quality of life. Our current model is far from real‐world use or clinical diagnoses; however, it is a first step in acknowledging the pathway forward for fMRI and deep learning's clinical applications. The next steps for implementation will require the upscaling of the initial baseline model (e.g., expanding the ASD dataset or accessing larger multiclass datasets), the external validation of the technique in clinical settings, and the refinement of the model to meet the needs of clinicians (e.g., automation and explainability). We hope this work can inspire other researchers to consider the clinical viability of their models and, one day, create an fMRI deep learning model that is generalizable, accurate, and economical for clinical AD diagnoses.

## Author Contributions

**Samuel L. Warren**: conceptualization, data curation, formal analysis, investigation, methodology, project administration, resources, software, validation, visualization, writing–original draft, writing–review and editing. **Ahmed A. Moustafa**: conceptualization, funding acquisition, supervision, validation, writing–review and editing.

## Ethics Statement

Ethics approval was received from Bond University prior to conducting this study.

## Conflicts of Interest

The authors declare no conflicts of interest.

### Peer Review

The peer review history for this article is available at https://publons.com/publon/10.1002/brb3.70427

## Data Availability

The data for this study was acquired from the ABIDE preprocessed repository and ADNI. This data is freely and openly available via their websites. ADNI data collection and sharing for this project was funded by ADNI (National Institutes of Health Grant U01 AG024904) and DOD ADNI (Department of Defence award number W81XWH‐12‐2‐0012). ADNI is funded by the National Institute on Aging, the National Institute of Biomedical Imaging and Bioengineering, and through generous contributions from the following: AbbVie, Alzheimer's Association; Alzheimer's Drug Discovery Foundation; Araclon Biotech; BioClinica, Inc.; Biogen; Bristol‐Myers Squibb Company; CereSpir, Inc.; Cogstate; Eisai Inc.; Elan Pharmaceuticals, Inc.; Eli Lilly and Company; EuroImmun; F. Hoffmann‐La Roche Ltd and its affiliated company Genentech, Inc.; Fujirebio; GE Healthcare; IXICO Ltd.; Janssen Alzheimer Immunotherapy Research & Development, LLC.; Johnson & Johnson Pharmaceutical Research & Development LLC.; Lumosity; Lundbeck; Merck & Co., Inc.; Meso Scale Diagnostics, LLC.; NeuroRx Research; Neurotrack Technologies; Novartis Pharmaceuticals Corporation; Pfizer Inc.; Piramal Imaging; Servier; Takeda Pharmaceutical Company; and Transition Therapeutics. The Canadian Institutes of Health Research is providing funds to support ADNI clinical sites in Canada. Private sector contributions are facilitated by the Foundation for the National Institutes of Health (www.fnih.org). The grantee organization is the Northern California Institute for Research and Education, and the study is coordinated by the Alzheimer's Therapeutic Research Institute at the University of Southern California. ADNI data are disseminated by the Laboratory for Neuro Imaging at the University of Southern California.
